# Race‐specific temporal trends of HPV‐related cancers in South Africa: An analysis of the South African National Cancer Registry, 2011–2022

**DOI:** 10.1002/ijc.70323

**Published:** 2025-12-31

**Authors:** Adino T. Tsegaye, Sizeka A. Mashele, Jaimie Z. Shing, Judith Mwansa‐Kambafwile, Aimée R. Kreimer, Carole Metekoua, Meredith S. Shiels, Mazvita Muchengeti

**Affiliations:** ^1^ National Cancer Institute Rockville Maryland USA; ^2^ National Cancer Registry National Health Laboratory Service Johannesburg South Africa; ^3^ Institute for Medical Epidemiology, Biostatistics and Informatics Martin‐Luther‐University Halle‐Wittenberg Halle Germany; ^4^ Swiss Tropical and Public Health Institute Allschwil Switzerland; ^5^ University of Basel Basel Switzerland; ^6^ School of Public Health University of the Witwatersrand Johannesburg South Africa; ^7^ School of Public Health University of Cape Town Cape Town South Africa; ^8^ South African DSI‐NRF Centre of Excellence in Epidemiological Modelling and Analysis Stellenbosch University Stellenbosch South Africa

**Keywords:** HPV‐related cancers, incidence rate, racial differences, South Africa, temporal trend

## Abstract

Evaluating trends in HPV‐related cancer rates by race is essential for identifying high‐risk populations and improving prevention efforts. Using 2011–2022 South African National Cancer Registry data, we analyzed age‐standardized incidence rates by race and sex across three periods (2011–2014, 2015–2018, 2019–2022) using linear regression. Significant increases were observed for oropharyngeal squamous cell carcinoma (SCC) among White females (*p* < .01), vulvar SCC among Asian (*p* < .01) and Black (*p* = .02) females, and anal SCC among Colored females and Black males (*p* < .01). Cervical carcinoma rates remained stable for most racial groups, except for the annual trends showing a 1.9% increase per year (95% CI = 1.0, 2.7) among White females. These findings suggest rising incidence rates for some HPV‐related cancers across racial groups in South Africa. Further research is needed to explore the constellation of risk factors contributing to these trends and to guide targeted interventions.

## Graphical abstract

What's new?

Racial disparities in trends of human papillomavirus (HPV)–associated cancers in South Africa have received limited attention. Here, the authors examined cancer incidence rates among South Africans, with stratification by race and sex, for different timeframes across 2011–2022. Significant increases were observed in oropharyngeal squamous cell carcinoma (SCC) among White females, vulvar SCC among Asian and Black females, and anal SCC among Colored females and Black males. Notably, for all timeframes, cervical cancer incidence was highest among Black women and increased relatively modestly among White women. The variations detected may reflect racial differences in HPV prevalence and healthcare access.

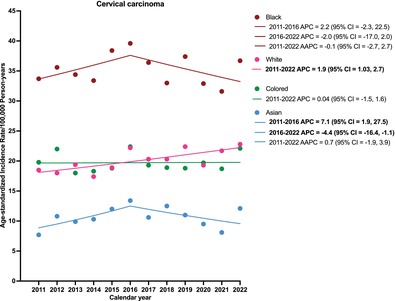

**Abbreviations**
AAPC: average annual percentage change
APC: annual percentage change
ASIR: age‐standardized incidence rate
CI: confidence interval
HPV: human papillomavirus
ICD: international classification of diseases
IR: incidence rate
LR: linear regression
NHLS: National Health Laboratory Services of South Africa
SCC: squamous cell carcinoma

## 1 INTRODUCTION

Racial differences in HIV and human papillomavirus (HPV) prevalence and socioeconomic status could lead to substantive differences in HPV‐associated cancer rates and trends over time in South Africa.^1^, ^2^, ^3^, ^4^ For instance, the prevalence of cervical HPV infection in South Africa ranges from 20% to 80%, varying by province, age group, and HIV status.^5^, ^6^, ^7^, ^8^ More than 55% of females aged 18–29 in South Africa have high‐risk HPV.^6^, ^9^ The prevalence of HIV among 15–49 year‐old Black females is 24%, which is 20 times higher than White and Asian South Africans and four times higher than the Colored (Mixed‐race) population.^1^, ^10^ Immunosuppression puts people with HIV at a higher risk of developing HPV‐related cancers than people without HIV.^11^, ^12^, ^13^, ^14^, ^15^ Socioeconomic status is closely linked with race, as Black South Africans persistently face elevated levels of poverty, unemployment, and restricted access to healthcare. These factors increase the risks associated with both HIV and HPV while simultaneously limiting opportunities for preventive care.^16^

Screening for cervical cancer using cytology and HPV vaccination of adolescent girls is the only available strategy for the prevention of HPV‐related cancers in South Africa. National surveys indicate that White and Asian females are more likely to participate in cervical cancer screening, with coverage estimates around 70%, compared to approximately 45%–55% among Black and Colored females. This disparity is attributed to variations in health literacy, affordability, and geographic access.^10^ In contrast, HPV vaccination coverage has been relatively higher among Black girls, with school‐based delivery in public schools achieving an 87% first‐dose coverage in the early years of the program, which began in 2014. Meanwhile, coverage is unknown among girls in private schools—attended more frequently by White and Asian children—who depend on private healthcare for vaccination.^17^, ^18^ The overlapping disparities in HIV, HPV, socioeconomic status, screening, and vaccination underscore the necessity of interpreting HPV‐related cancer risk and prevention strategies within the racially stratified public health context of South Africa.

Unique trends were reported in our recent evaluation of the incidence of HPV‐related cancers from 2011 to 2021 using the South African National Cancer Registry data. Specifically, oropharyngeal cancer incidence plateaued while cervical cancer incidence declined in 2016–2021. Rates of vaginal, vulvar, and anal cancers among women and penile and anal cancers among men increased.^19^ There was variation in HPV‐related cancer incidence rates by race, with Black people having higher incidence rates, but the extent to which these trends vary over time by race has not been thoroughly investigated. It is imperative to characterize and compare trends in HPV‐related cancer rates over time by race to inform cancer prevention efforts and better tailor interventions.^19^, ^20^

## 2 MATERIALS AND METHODS

In this study, we analyzed 2011–2022 South African National Cancer Registry de‐identified data to estimate race‐specific trends of HPV‐related cancer rates, including cervical carcinoma and vulvar, vaginal, penile, oropharyngeal, and anal squamous cell carcinomas (SCCs) based on the International Classification of Diseases (ICD‐0‐3) codes. The South African National Cancer Registry procedures are described in full elsewhere.^21^ Briefly, the South African National Cancer Registry is a division of the National Health Laboratory Services (NHLS) of South Africa. Since 1986, the National Cancer Registry has collected all laboratory diagnosed cancers from private and public health‐care laboratories in South Africa by extracting demographic and tumor data from pathology reports including sex (female or male), race (Asian, Black, Colored, or White), date of diagnosis, year of diagnosis, cancer site, and cancer histology including all cancer morphology subtypes.^22^ Public and private laboratories report any cancer incidence through the Corporate Data Warehouse of the NHLS where the National Cancer Registry extracts cancer data. In 2011, Regulation 380 of the National Health Act of 2003 made cancer a reportable disease in South Africa. Thus, cancer incidence reporting after this period is considered robust and comprehensive.

Mid‐year national population estimates from Statistics South Africa were used to estimate person‐time, stratified by sex (female, male), year, age (5‐year intervals), and race (Black, White, Colored, Asian). The term “Colored” is used in South Africa to refer to mixed‐race individuals. We calculated IRs per 100,000 person‐years and age‐standardized to the 1960 Segi world standard population for each cancer type stratified by race and sex (for anal and oropharyngeal SCC) and estimated temporal trends. We assessed trends in age‐standardized IRs across year periods using a linear regression model, treating calendar periods as continuous. We estimated age‐standardized incidence rates (ASIRs) in 4‐year calendar periods (2011–2014, 2015–2018, and 2019–2022). Because cervical carcinoma has more cases, we also estimated the annual ASIR in cervical carcinoma and annual percentage changes (APCs) and average annual percentage changes (AAPCs) using Joinpoint regression. We used SEER*stat to estimate ASIRs, Joinpoint software for calculating the APC and AAPCs, and R version 4.4.1 for fitting the linear regression model. *p*‐trends <.05 for the changes in IRs over time were considered statistically significant.

## 3 RESULTS

**FIGURE 1:**
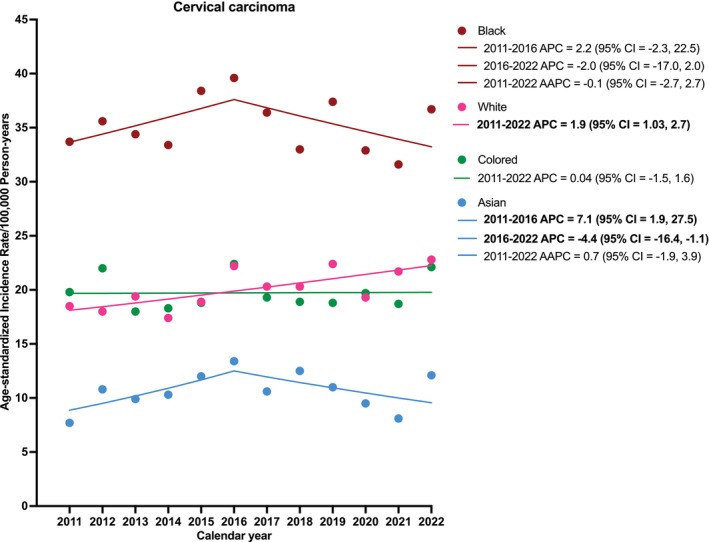
Racial trend of cervical carcinoma in South Africa (2011–2022). AAPC, average annual percent change; APC, annual percent change.

Between 2011 and 2022, ASIRs of cervical carcinoma across calendar periods were stable for all racial groups (Table 1). However, in the Joinpoint analysis, which assessed trends across individual calendar years, cervical carcinoma IRs increased among White females on average by 1.9% annually (95% CI = 1.03, 2.7), from 18.5 in 2011 to 22.8 per 100,000 person‐years in 2022. Among Asian females, cervical carcinoma incidence increased by 7.1% annually from 7.1 in 2011 to 13.4 per 100,000 person‐years in 2016 (95% CI = 1.9, 27.5) but significantly decreased by 4.4% annually from 2016 to 2022 (95% CI = −16.4, −1.1) (Figure 1). Although not statistically significant, the trend among Black females was similar to Asian females. Annual cervical carcinoma rates among Colored females were stable over time.

**TABLE 1:** Description of case counts and incidence rates by race for HPV‐related cancers in South Africa—2011–2022.

Sex	Cancer type		Black		White		Colored		Asian	
		Year	Count	IR (95% CI)	Count	IR (95% CI)	Count	IR (95% CI)	Count	IR (95% CI)
Female	Cervical carcinoma	2011–2014	17,580	34.3 (33.8, 34.8)	1679	18.3 (17.4, 19.2)	1389	19.5 (18.5, 20.6)	232	9.7 (8.5, 11.0)
		2015–2018	21,075	36.8 (36.3, 37.3)	1886	20.4 (19.4, 21.4)	1559	19.8 (18.8, 20.8)	315	12.1 (10.8, 13.5)
		2019–2022	21,993	34.6 (34.1, 35.1)	1937	21.5 (20.5, 22.6)	1680	19.9 (18.9, 20.9)	295	10.2 (9.0, 11.4)
		*p*‐value		.93		.11		.18		.87
	Oropharyngeal SCC	2011–2014	65	0.1 (0.1, 0.2)	65	**0.6 (0.4, 0.7)**	39	0.6 (0.4, 0.8)	7	0.3 (0.1, 0.6)
		2015–2018	86	0.2 (0.1, 0.2)	88	**0.7 (0.6, 0.9)**	60	0.8 (0.6, 1.0)	6	0.2 (0.1, 0.5)
		2019–2022	77	0.1 (0.1, 0.2)	89	**0.8 (0.6, 1.0)**	54	0.6 (0.5, 0.8)	7	0.2 (0.1, 0.5)
		*p*‐value		1.00		**<.01**		1.00		.33
	Vulvar SCC	2011–2014	830	**1.4 (1.3, 1.5)**	195	1.6 (1.4, 1.9)	101	1.4 (1.2, 1.8)	31	**1.3 (0.9, 1.8)**
		2015–2018	1568	**2.4 (2.3, 2.5)**	270	2.3 (2.0, 2.6)	113	1.4 (1.2, 1.7)	43	**1.5 (1.1, 2.0)**
		2019–2022	2394	**3.3 (3.2, 3.5)**	249	2.1 (1.9, 2.5)	167	2.0 (1.7, 2.3)	51	**1.7 (1.2, 2.2)**
		*p*‐value		.**02**		.51		.33		**<.01**
	Vaginal SCC	2011–2014	341	0.7 (0.6, 0.7)	43	0.4 (0.3, 0.5)	33	0.5 (0.3, 0.6)	11	0.4 (0.2, 0.8)
		2015–2018	491	0.9 (0.8, 0.9)	66	0.6 (0.4, 0.8)	33	0.4 (0.3, 0.6)	16	0.6 (0.3, 0.9)
		2019–2022	509	0.8 (0.7, 0.9)	71	0.6 (0.5, 0.8)	59	0.7 (0.5, 0.9)	16	0.5 (0.3, 0.8)
		*p*‐value		.67		.33		.55		.67
	Anal SCC	2011–2014	200	0.4 (0.3, 0.4)	71	0.6 (0.5, 0.8)	24	**0.4 (0.2, 0.5)**	6	0.2 (0.1, 0.5)
		2015–2018	408	0.6 (0.6, 0.7)	80	0.7 (0.5, 0.8)	35	**0.5 (0.3, 0.6)**	6	0.2 (0.1, 0.5)
		2019–2022	624	0.9 (0.8, 1.0)	85	0.7 (0.6, 0.9)	51	**0.6 (0.4, 0.8)**	10	0.4 (0.2, 0.7)
		*p*‐value		.07		.33		**<.01**		.33
Male	Oropharyngeal SCC	2011–2014	327	1.0 (0.9, 1.2)	214	2.1 (1.8, 2.4)	133	2.5 (2.1, 3.0)	21	1.1 (0.7, 1.6)
		2015–2018	352	1.0 (0.9, 1.1)	264	2.5 (2.2, 2.8)	156	2.7 (2.3, 3.2)	18	0.8 (0.5, 1.2)
		2019–2022	408	1.0 (0.9, 1.1)	272	2.4 (2.1, 2.7)	172	2.5 (2.2, 2.9)	20	0.8 (0.5, 1.2)
		*p*‐value		.79		.49		1.00		.33
	Penile SCC	2011–2014	446	1.1 (1.0, 1.2)	85	0.8 (0.6, 1.0)	34	0.7 (0.5, 1.0)	16	0.8 (0.5, 1.4)
		2015–2018	599	1.4 (1.3, 1.5)	123	1.1 (0.9, 1.4)	46	0.8 (0.6, 1.1)	19	0.8 (0.5, 1.3)
		2019–2022	1004	2.1 (1.9, 2.2)	138	1.2 (1.0, 1.5)	84	1.3 (1.0, 1.6)	20	0.8 (0.5, 1.2)
		*p*‐value		.14		.18		.23		.33
	Anal SCC	2011–2014	100	**0.2 (0.2, 0.3)**	47	0.5 (0.3, 0.6)	22	0.4 (0.2, 0.6)	6	0.3 (0.1, 0.7)
		2015–2018	187	**0.4 (0.3, 0.4)**	51	0.5 (0.4, 0.7)	22	0.4 (0.2, 0.6)	7	0.3 (0.1, 0.6)
		2019–2022	343	**0.6 (0.6, 0.7)**	57	0.5 (0.4, 0.7)	42	0.6 (0.4, 0.8)	10	0.4 (0.2, 0.7)
		*p*‐value		**<.01**		.33		.33		.33

*Note*: There were 335 cases with unknown race from all anatomical sites and both sexes. The bold values indicates statistically significant findings.

Abbreviations: APC, annual percentage change; CI, confidence interval; IR, incidence rate; LR, linear regression; SCC, squamous cell carcinomas.

For non‐cervical HPV‐driven cancers, oropharyngeal SCC ASIRs per 100,000 person‐years increased only among White females (from 0.6 in 2011–2014 to 0.8 in 2018–2022, *p*‐trend <.01) (Table 1). Vulvar SCC ASIRs increased among Black females from 1.4 in 2011–2014 to 3.3 in 2018–2022 (*p*‐trend = .02) and Asian females from 1.3 in 2011–2014 to 1.7 in 2018–2022 (*p*‐trend <.01) with no statistically significant change among White and Colored females. Vaginal SCC ASIRs did not change significantly in any racial group. Anal SCC IRs increased among Colored females (IR = 0.4 in 2011–2014 to 0.6 in 2018–2022; *p*‐trend <.01), and among Black females (IR = 0.4 in 2011–2014 to 0.9 in 2018–2022; *p*‐trend = .07), with no statistically significant change in other racial groups. Among males, IRs for oropharyngeal and penile SCC did not change significantly in any racial group. Anal SCC IRs increased only among Black males from 0.2 in 2011–2014 to 0.6 in 2018–2022 (*p*‐trend <.01).

## 4 DISCUSSION

In our prior study of trends in HPV‐related cancers in South Africa aggregated across races,^19^ we observed an increasing trend for vulvar, vaginal, and anal SCC among females and penile and anal SCC among males. In the current analysis, we found that there are distinct racial patterns within these trends. There were significant increases in rates of oropharyngeal SCC among White females, vulvar SCC among Asian and Black females, and anal SCC among Colored females and Black males. Specifically for cervical cancer, incidence was highest among Black women across the time period, with no significant change over time, while White women showed a modest but significant increase. Among Asian women, cervical cancer incidence initially rose between 2011 and 2016, followed by a slight decrease thereafter. Colored women displayed relatively stable trends. These observed differences could reflect racial differences in access to healthcare and possible heterogeneity in HIV and HPV prevalence.

South Africa has had a free‐of‐charge cytology‐based cervical screening program since 2000, providing three Pap smears in a woman's lifetime, starting at age 30 with 10‐year intervals for the general population and screening at HIV diagnosis and every 3 years for women with HIV. Despite this policy, screening coverage remains suboptimal. Nationally, only around 52% of eligible women report ever having had a Pap smear, and coverage is lowest among Black and Colored women compared to White and Asian women.^10^ The lack of a substantial decline in cervical cancer incidence across all racial groups, including those with historically better healthcare access, highlights structural weaknesses of the program, such as late initiation, long intervals between tests, poor follow‐up of abnormal results, and limited reach in rural or under‐resourced areas.

Among Asian and Black women, cervical cancer rates increased from 2011 to 2016, and then decreased in recent years, which may be related to changes in cancer‐related policies such as cancer reporting, cervical cancer screening, COVID‐19 regulations and HIV test and treat, resulting in changes in screening rates and healthcare uptake. The absence of a statistically significant AAPC among Asian women suggests these fluctuations should be interpreted cautiously. These trends reflect the need for a thorough evaluation of the screening program, which failed to bring a meaningful decline in the incidence of cervical cancer across all racial groups.

The increasing IR of oropharyngeal SCC among White females could be partly due to increases in oral HPV infection rates because of higher prevalence of oral sex practices,^23^ as well as increased smoking and alcohol drinking. Vulvar cancer rates are increasing in Black and Asian women, but not in White and Colored women. The observed racial differences in vulvar cancer align with known etiological pathways. Only a subset of vulvar cancers is HPV‐associated, typically occurring in younger women, while HPV‐independent vulvar cancers tend to present at older ages. The increase among younger Black women is likely related to high HIV prevalence, which amplifies HPV persistence and progression.^19^ By contrast, the increase among Asian women was primarily observed in older age groups, consistent with HPV‐independent vulvar carcinogenesis. These divergent patterns underscore the complexity of vulvar cancer etiology and the importance of considering both viral and non‐viral pathways. Similarly, the rising incidence of anal SCC among Black and Colored women and Black men likely reflects the combined effect of a high HIV burden in these groups and the absence of routine anal cancer screening in South Africa. HIV‐associated immunosuppression increases susceptibility to persistent anal HPV infection, yet no formal program exists for early detection and prevention.^1^, ^3^

However, additional studies on risk factors and underlying mechanisms of each cancer type may help to decipher the variabilities across racial groups and inform cancer prevention efforts. HPV vaccination represents a critical preventive tool. South Africa introduced a national, school‐based HPV vaccination program in 2014, targeting grade 4 girls (aged 9–10 years) in public schools with a two‐dose schedule at the early stages of the program. Initial coverage was high, with first‐dose uptake of 86%–88% and second‐dose completion around 72%–80%. However, recent reports suggest declining coverage, especially after COVID‐19, with disruptions in school‐based delivery and logistical challenges. Furthermore, racial and socioeconomic disparities persist: Black girls, who are more likely to attend public schools, have benefited most from the national program, whereas White and Asian girls, more commonly enrolled in private schools, must access vaccination through private healthcare, which may lead to lower coverage. The long‐term population‐level impact of vaccination is therefore uncertain and requires sustained program monitoring.

## AUTHOR CONTRIBUTIONS

**Adino T. Tsegaye:** Conceptualization; methodology; software; data curation; investigation; validation; formal analysis; visualization; writing – original draft. **Sizeka A. Mashele:** Conceptualization; data curation; writing – original draft; writing – review and editing. **Jaimie Z. Shing:** Conceptualization; methodology; investigation; software; supervision; writing – review and editing. **Judith Mwansa‐Kambafwile:** Project administration; data curation; writing – review and editing. **Aimée R. Kreimer:** Writing – review and editing; conceptualization; investigation; methodology; supervision. **Carole Metekoua:** Data curation; writing – review and editing. **Meredith S. Shiels:** Conceptualization; investigation; methodology; supervision; writing – review and editing. **Mazvita Muchengeti:** Conceptualization; methodology; investigation; project administration; data curation; supervision; writing – review and editing.

## CONFLICT OF INTEREST STATEMENT

The authors declare no conflicts of interest.

## ETHICS STATEMENT

The study was approved by the Division of Cancer Epidemiology and Genetics at the US National Cancer Institute and by the division of the South African National Cancer Registry at the National Health Laboratory Services of South Africa. It complies with the general data protection regulations, and the use of registry‐based data for scientific studies in South Africa does not require participant consent.

## Data Availability

The data underlying this article are available from the South African National Cancer Registry database https://www.nicd.ac.za/centres/national-cancer-registry/. Further information is available from the corresponding author upon reasonable request.
